# Recent Studies of Hypnotism

**Published:** 1887-12

**Authors:** 


### RECENT STUDIES OF HYPNOTISM.

A letter from Paris, in the *New York Medical Journal*, for March 19th last, treats of those unexplained phenomena which, under the name of mesmerism, remained so long the objects of simple curiosity and amusement, but which are now being submitted to rigid scientific investigation, disclosing how vast the field is and how many medical and social problems the study raises Whether or not the use of magnets for transferring such symptoms from one patient to another will ever amount to anything of real value only time can show. At any rate, hypnotism, or "suggestion" to the hypnotized patient, is a subject of medical investigation that has attracted a great deal of attention recently among all classes in Paris, owing to the fact that its medico-legal aspect has come under examination. It is easy to see that it is possible for an individual to acquire an unlimited power of action upon another, so as to be able to impose his will upon him and cause him to do whatever he likes. If this can be proved, the sphere of legal responsibility will he greatly modified. Public opinion in France has been much moved by these matters, and the government was urged to appoint a committee to examine into the question. This was done, and the committee held weekly sittings at the Saltpetriere Hospital. The committee was composed of magistrates and professors of mental medicine, with Dr. Brouardel, the Paris professor of legal medicine. The principal questions examined into were the following: Can a person cause another, when in a state of hypnotism, to sign receipts for money not received? Can a person, in the same state, be forced, against his or her will, to draw a will in favor of anybody?

The mode of experimentation was as follows: A female patient, Mademoiselle A., is forced into the lethargic sleep by pressure on a suggested hypnotic point, when by slight friction on the forehead, she passes into the somnambulistic state. Professor Brouardel then approaches her and asks if she will accept the loan of fifty francs. At first she refuses, but, on the suggestion being forced upon her, she gradually weakens, and finally consents to accept the offer. A stamped receipt is then drawn up with every possible legal precaution, and the patient herself is quite anxious that there should be no mistake about it. She then signs it, and Dr. Brouardel puts it into his pocket, but does not offer to give her the money. She is then awakened and acknowledges that the receipt is signed by her, but cannot remember under what circumstances she was induced to sign it, or whether or not she got the money. Legally the receipt is quite valid, and, according to the present law, the holder could collect payment if the signer had any means of payment. In regard to the second matter, that of compelling a person to draw up a will in a certain way, the experiment was equally successful. Mademoiselle B. is plunged into the hypnotic state, and Dr. Babinski tells her it is absolutely necessary for her to make her will at once, and in his favor. She objects at first. saying that she is too young to die, etc. This lasts about ten minutes, and she goes on to say also that she wishes to give her property to her mother and other relations, but after persistent persuasion and keeping up the suggestion that it is better to give everything to Dr. Babinski, she at last begins to weaken and finally accepts the proposition, saying that her property consists of about thirty francs that she has saved, and that she has a ring, a brooch, and a pair of earrings. All this, her sole property, she then begins to bequeath to Dr. Babinski, and the next Thursday is appointed for the signing of the will. A notary is to draw up the document and she will sign it. Moreover, Dr. Babinski suggests to her to say nothing about it to any one in the meantime, and to say when asked that she acted on her own free will and consent, and that she was not forced to the act by anybody. The appointed day arrives, and it is noticed that the girl has been rather fidgety and nervous since early m rning, and says she has something to do, but does not remember what it is. On being put into the somnambulistic state, however, she remembers her promise, and when one of the bystanders is introduced as the lawyer, she immediately draws up her will and gives all that she has to the doctor. This is duly witnessed, and then the lawyers of the committee question her as to whether she has heen urged to the act. She replies that she has done it all of her own free will; that she knows that she has a poor family, but she would rather give everything she has to Dr. Babinski. She says, however, that she is obliged to do so, but when asked for what reason, cannot tell. When she is awakened, she repeats the same story.

These experiments prove the legal irresponsibility of these patients, for it is obvious that they can be made to commit many acts without knowing why it is that they are compelled, as it were, to do them. In his faculty lectures, this year, on rape, etc., referring to the question of the bearing of hypnotism on his subject, Brouardel said that he was not disposed to believe that suggestion could be used in the commission of criminal acts against the person. To be sure, the possibility of criminal connection in the mesmerized state was not exactly denied, but it must be taken into account that the female subjects must be entirely consenting parties before they are put into the sleep, and that it is probably impossible to get them into the sleep without their consent. Besides, there is no evidence that coitus can be accomplished without the patient's knowledge; indeed, it is often astonishing how much they do know and hear when in the trance-like state, as is frequently proved in the hospital when some one is rash enough to make depreciating remarks, and is told of it pretty sharply when the patient awakes.

On the other hand, it seems not unlike, if we may trust a cable dispatch from Paris to the *Herald*, that the study of hypnotism will prove a powerful aid to legal procedure, inasmuch as by sending criminals to sleep and dragging their secret from them while under this influence, there would be little fear of judges condemning the innocent for the guilty. A theft in the hospital was found out in this way by Dr. Marie, for many years Dr. Charcot's assistant. The subject refused at first to tell where the stolen object was concealed. After a little diplomacy, however, on the part of the young doctor, who told the sleeping girl he was the young man from whom the card case had been taken and not to fear telling him where it was, she gave the detailed account of having stolen it, and told where the card case was to be found. Dr. Marie immediately went to the spot indicated, and, sure enough, there was the stolen article.

One of the curious sides of this matter is shown in the religious journal, *L'Univers*, which seems to see a terrible heresy in the study of hypnotism, and denounces the new science as "dangerous to morality." In his studies, M. Charcot called in the aid of instantaneous photography, and he has taken his patients in every phase and attitude of their complaints. Afterward, when the history of these maladies was hunted up, it was found that these attitudes were precisely those represented in certain ancient works of art. All who know M. Charcot know that he is something of an artist himself. He has a great taste for art, and every year, when traveling, he has visited old churches and museums. He has been struck at finding that old church paintings, portraying the lives of saints and those who were supposed to be "possessed," represented exactly the appearance that instantaneous photography revealed in his hysterical patients. This idea was followed up, and long search proved that paintings by Andrea del Sarte, Rubens, Rosselli, Van Noort, and many others of the old masters were simply copies from nature, faithfully representing the convulsions of hysterical men and women. Some very curious examples of these "miracles" were certainly only manifestations of St. Vitus's dance or hysteria. So we fear that another of the world's cherished ideas is being decidedly undermined—hence the wrath of the pious sheet against M. Charcot and his fellow-workers.—*New York Medical Times.*

